# Cervical cancer in the modern era: cutting-edge strategies for diagnosis and treatment

**DOI:** 10.1007/s11604-025-01897-0

**Published:** 2025-10-28

**Authors:** Tsukasa Saida, Takashi Saitoh, Ayumi Shikama, Michitaka Hayashi, Taichi Ishikawa, Mami Iima, Takahito Nakajima, Shinji Naganawa

**Affiliations:** 1 Department of Radiology, Institute of Medicine, University of Tsukuba, 1-1-1 Tennodai, Tsukuba, Ibaraki 305-8575 Japan; 2 Department of Radiation Oncology, Proton Medical Research Center, Institute of Medicine, University of Tsukuba, 1-1-1 Tennodai, Tsukuba, Ibaraki 305-8575 Japan; 3 Department of Obstetrics and Gynecology, Institute of Medicine, University of Tsukuba, 1-1-1 Tennodai, Tsukuba, Ibaraki 305-8575 Japan; 4 Department of Diagnostic and Interventional Radiology, University of Tsukuba Hospital, 2-1-1 Amakubo, Tsukuba, Ibaraki 305-8576 Japan; 5 Department of Radiology, Nagoya University Graduate School of Medicine, 65 Tsurumai-cho, Showa-ku, Nagoya, Aichi 466-8550 Japan

**Keywords:** Human papillomavirus, Magnetic resonance imaging, Artificial intelligence, Radiotherapy, Fertility-sparing treatment

## Abstract

Cervical cancer remains a major cause of mortality among women worldwide, highlighting the need for advances in diagnostic and therapeutic strategies. This review provides a comprehensive overview of recent developments in histopathological classification based on human papillomavirus association, as well as progress in imaging and treatment approaches supported by current research. Emerging technologies, such as artificial intelligence-assisted imaging, targeted molecular therapies, and personalized radiotherapy, hold substantial promise for improving patient outcomes. These innovations offer new possibilities for precision medicine in cervical cancer care, and their current applications are discussed in this review.

## Introduction

In 2022, approximately 662,301 new cases and 348,874 deaths from uterine cervical cancer were reported worldwide, highlighting a significant global health burden [1]. The disease is most prevalent in low- and middle-income countries, primarily because of limited access to screening and treatment. Risk factors include early sexual activity, multiple sexual partners, immunosuppression, smoking, and a lack of regular screening [2]. Infection with high-risk types of human papillomavirus (HPV), particularly HPV-16 and HPV-18, is the primary cause. The World Health Organization (WHO) has launched a global initiative to eliminate cervical cancer. HPV vaccination, particularly among adolescents before their sexual debut, has significantly reduced infection rates. Nevertheless, cervical cancer poses major public health challenges worldwide. Histologically, most cervical cancers are squamous cell carcinomas (SCC) followed by adenocarcinomas (AC). In contemporary classification, both are now recognized to include HPV-associated and HPV-independent subtypes [2].

This review examined the histopathological characteristics and HPV-based classification of cervical cancer, with a particular focus on the role of imaging in distinguishing subtypes. Furthermore, this review highlights recent advances in diagnostic imaging and state-of-the-art therapeutic strategies.

## Histology

A summary of the WHO 2020 classification of uterine cervical cancers is presented in Table 1.

**Table 1:** Summary of the World Health Organization 2020 classification of uterine cervical cancers

Category	Tumor entity	HPV status	Characteristics
SCC			
	HPV-associated	+	Most common, 80–90% of cervical cancers
	HPV-independent	−	5–7% of SCCs
AC			
	HPV-associated	+	10–25% of cervical cancers in developed countries, including usual type (~ 75%) and mucinous type (~ 10%)
	HPV-independent, gastric type	−	10–15% of ACs, associated with LEGH
	HPV-independent, clear cell type	−	3–4% of ACs, associated with in utero exposure to diethylstilbestrol
	HPV-independent, mesonephric type	−	Rare, < 0.1% of ACs, arising from mesonephric remnants within the cervical wall
Adenosquamous		+	5–6% of cervical cancers, exhibiting both squamous and glandular differentiation

*SCC* squamous cell carcinoma, *AC* adenocarcinoma, *HPV* human papillomavirus, *LEGH* lobular endocervical glandular hyperplasia

### Squamous cell carcinoma, HPV-associated

Most cervical cancers (80–90%) are SCC and associated with HPV infection. Twelve HPV types are classified by the WHO, and HPV-16 and HPV-18 are responsible for 70% of all SCCs. The progression of high-grade lesions to SCC requires 20–30 years. Diagnosis typically occurs in the 60 s in high-income countries [2].

**Fig. 1:**
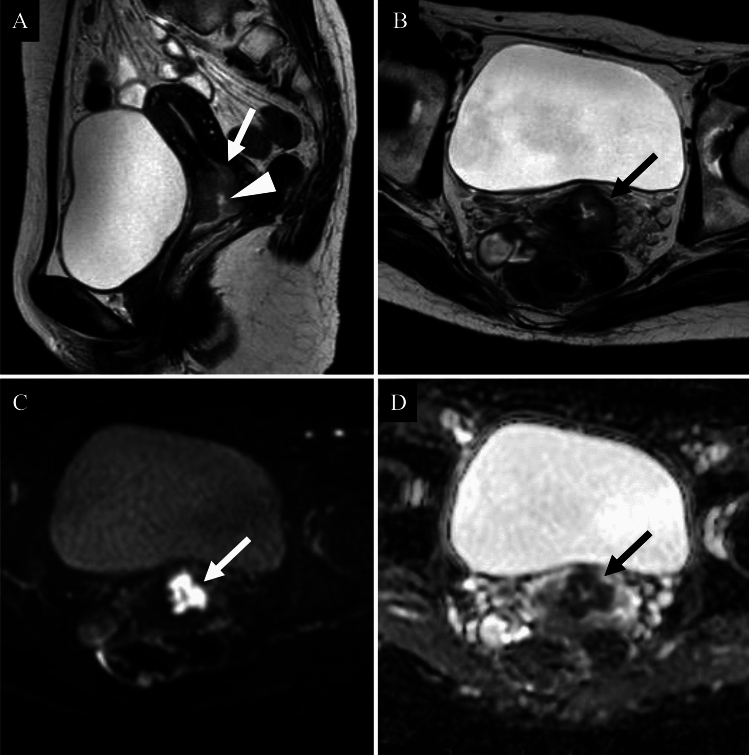
Squamous cell carcinoma of the uterine cervix in a woman in her fifties. **A** Sagittal T2-weighted image; **B** Axial T2-weighted image; **C** Axial diffusion-weighted image; **D** Axial apparent diffusion coefficient map. A cervical mass exhibiting high signal intensity relative to the surrounding stroma on T2-weighted image is identified (**A**, **B**: arrows). A central depression consistent with ulceration is observed (**A**: arrowhead). The lesion shows a markedly high signal on diffusion-weighted image (**C**: arrow) and significantly reduced apparent diffusion coefficient values (**D**: arrow), with a mean ADC of 0.783 × 10⁻^3^ mm^2^/s. The tumor is confined to the uterus, with no evidence of parametrial invasion. A diagnosis of FIGO 2018 stage IIA1 HPV-associated squamous cell carcinoma was confirmed following surgery

In the 2018 International Federation of Gynaecology and Obstetrics (FIGO) staging system for cervical cancer (Table 2). MRI is recognized as a valuable adjunct for assessing local tumor extent and lymph node (LN) status, although clinical staging remains the foundation. According to the ACR Appropriateness Criteria, MRI without and with IV contrast is categorized as “usually appropriate” [3]. On T2-weighted imaging, tumors appear hyperintense compared to the low-signal cervical stroma. Diffusion-weighted imaging (DWI) improves lesion visibility and accuracy in detecting tumors and assessing parametrial invasion [4, 5] (Fig. 1). In contrast, according to the European Society of Urogenital Radiology (ESUR) guidelines, contrast-enhanced MRI offers limited additional value for assessing parametrial invasion and is not considered essential. Dynamic contrast-enhanced (DCE) MRI is used primarily in research settings. Although it has been investigated for the evaluation of small tumors after conization, its clinical application remains limited.

**Table 2:** FIGO 2018 staging uterine cervical cancer

Stage	Description
I	Carcinoma confined to the cervix (including microscopic and clinically visible lesions)
IA	Invasive carcinoma diagnosed only by microscopy
IA1	Stromal invasion ≤ 3 mm in depth and ≤ 7 mm in horizontal spread
IA2	Stromal invasion > 3 mm and ≤ 5 mm in depth and ≤ 7 mm in horizontal spread
IB	Clinically visible lesion or microscopic lesion > IA2, confined to the cervix
IB1	Tumor ≤ 2 cm in greatest dimension
IB2	Tumor > 2 cm and ≤ 4 cm
IB3	Tumor > 4 cm
II	Tumor invades beyond the cervix but not to the pelvic wall or lower third of the vagina
IIA	Involvement limited to the upper two-thirds of the vagina, no parametrial invasion
IIA1	Tumor ≤ 4 cm
IIA2	Tumor > 4 cm
IIB	Parametrial invasion but not up to the pelvic wall
III	Tumor extends to the pelvic wall, involves lower third of the vagina, or causes hydronephrosis or non-functioning kidney
IIIA	Involves lower third of the vagina
IIIB	Extension to the pelvic wall and/or hydronephrosis/non-functioning kidney
IIIC	Pelvic and/or para-aortic lymph node metastasis (detected radiologically or pathologically)
IIIC1	Pelvic lymph node involvement
IIIC2	Para-aortic lymph node involvement
IV	Tumor invades beyond the true pelvis or involves (biopsy-proven) mucosa of bladder or rectum
IVA	Invasion of adjacent pelvic organs (bladder/rectum)
IVB	Distant metastasis (e.g., lung, liver, bone)

### Squamous cell carcinoma, HPV-independent

Approximately 5–7% of cervical SCCs are HPV-independent, typically occurring in older women, often in their 70 s. Although these tumors macroscopically resemble HPV-associated SCCs, they are more often diagnosed at advanced stages with higher LN metastasis rates and poorer survival [2].

Although the imaging features are similar, a recent study reported that radiomics models using features from T2-weighted imaging, contrast-enhanced T1-weighted imaging, and their combination achieved up to 95% accuracy in predicting carcinogenic HPV status [6].

### Adenocarcinoma, HPV-associated

HPV-associated AC is a glandular tumor exhibiting invasive and/or expansile exophytic growth, typically presenting at a younger age (40–42 years) than HPV-independent AC. HPV types 16, 18, and 45 account for approximately 95% of the cases. Histologically, HPV-associated AC includes the usual (most common, including the villoglandular variant) and mucinous subtypes (not otherwise specified, intestinal, signet ring cell, and stratified mucin producing carcinoma) [2]. Villoglandular carcinoma, a rare subtype (3.7–4.8% of ACs), affects younger women. It generally has a favorable prognosis, and fertility-sparing surgery may be appropriate in the early stages [2, 7, 8].

**Fig. 2:**
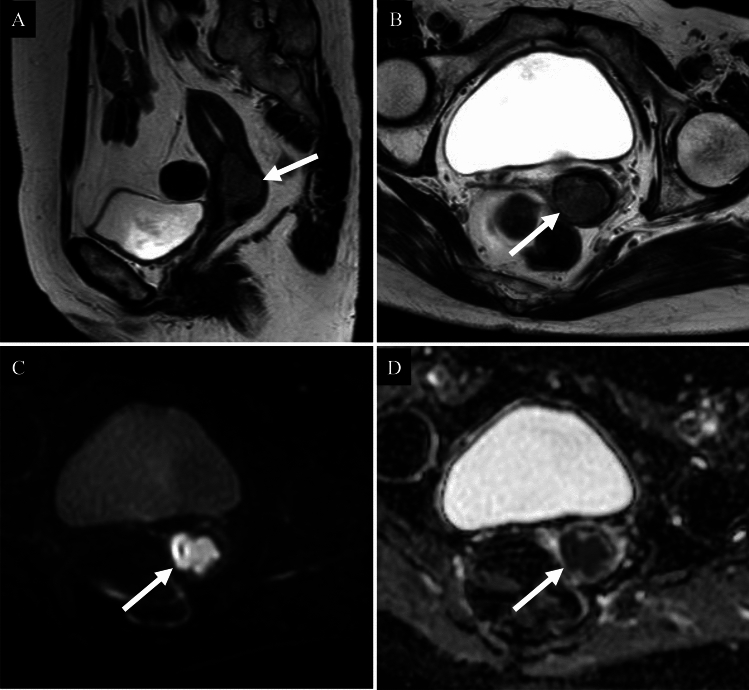
Usual type adenocarcinoma of the uterine cervix in a woman in her sixties. **A** Sagittal T2-weighted image; **B** Axial T2-weighted image; **C** Axial diffusion-weighted image; **D** Axial apparent diffusion coefficient map. A well-defined expansile cervical mass with high signal intensity relative to the surrounding stroma is observed on T2-weighted image (**A**, **B**: arrows). The lesion demonstrates a markedly high signal on diffusion-weighted image (**C**: arrow) and significantly reduced apparent diffusion coefficient values (**D**: arrow), with a mean ADC of 0.674 × 10⁻^3^ mm^2^/s. The posterior right cervical stroma appears thin but preserved, with no imaging evidence of parametrial infiltration. Pathological examination confirmed FIGO 2018 stage IB1 usual type HPV-associated adenocarcinoma

**Fig. 3:**
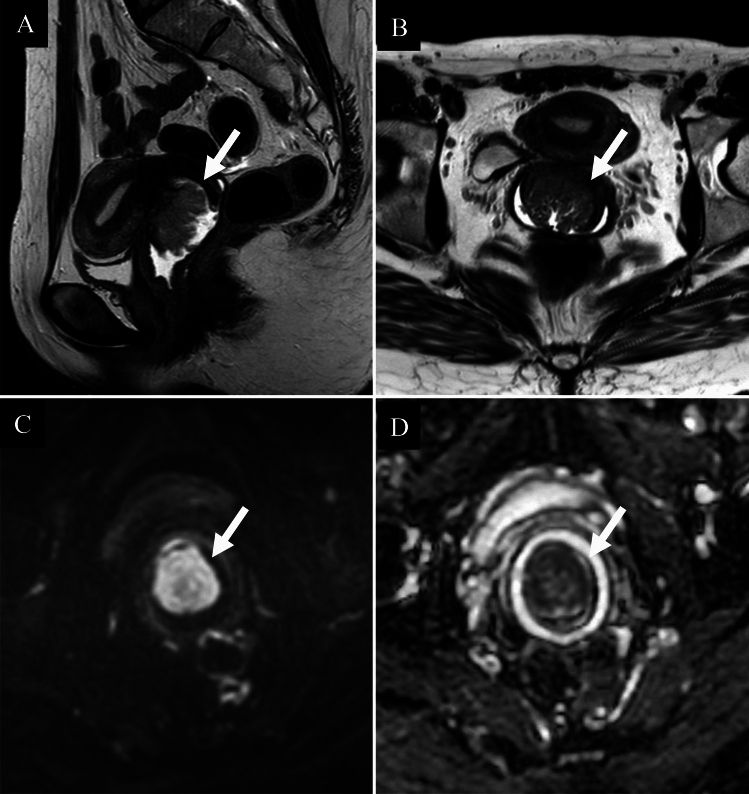
Villoglandular carcinoma of the uterine cervix in a woman in her thirties. **A** Sagittal T2-weighted image; **B** Axial T2-weighted image; **C** Axial diffusion-weighted image; **D** Axial apparent diffusion coefficient map. An exophytic, villous-papillary mass protruding from the uterine cervix demonstrates a “fern leaf-like” architecture on T2-weighted images (**A**, **B**: arrows) and restricted diffusion on diffusion-weighted imaging (**C**, **D**: arrows), with a mean ADC of 1.006 × 10⁻^3^ mm^2^/s. Pathological examination confirmed FIGO 2018 stage IB1 villoglandular carcinoma

Most comparative MRI studies have focused on all ACs versus SCC [9], or usual type AC versus gastric type AC (GAS) [9, 10]. However, no study has specifically compared usual type AC with SCC and differentiation using imaging alone is difficult (Fig. 2). Villoglandular carcinoma typically shows an exophytic, villous-papillary mass with a “fern leaf-like” architecture and restricted diffusion on DWI [7, 8] (Fig. 3).

### Adenocarcinoma, HPV-independent, gastric type

GAS accounts for 10–15% of all cervical ACs and is not associated with high-risk HPV infection [2]. This category includes minimal deviation AC, historically termed adenoma malignum, which is more aggressive than HPV-associated types, with greater rates of destructive stromal invasion, extrauterine spread, and diagnosis at an advanced stage. Both disease-free and overall survival rates are markedly poorer. Lobular endocervical glandular hyperplasia (LEGH), often with pyloric gland differentiation, is considered a potential precursor and is frequently associated with watery or mucoid vaginal discharge and is prevalent in patients with Peutz–Jeghers syndrome [2].

**Fig. 4:**
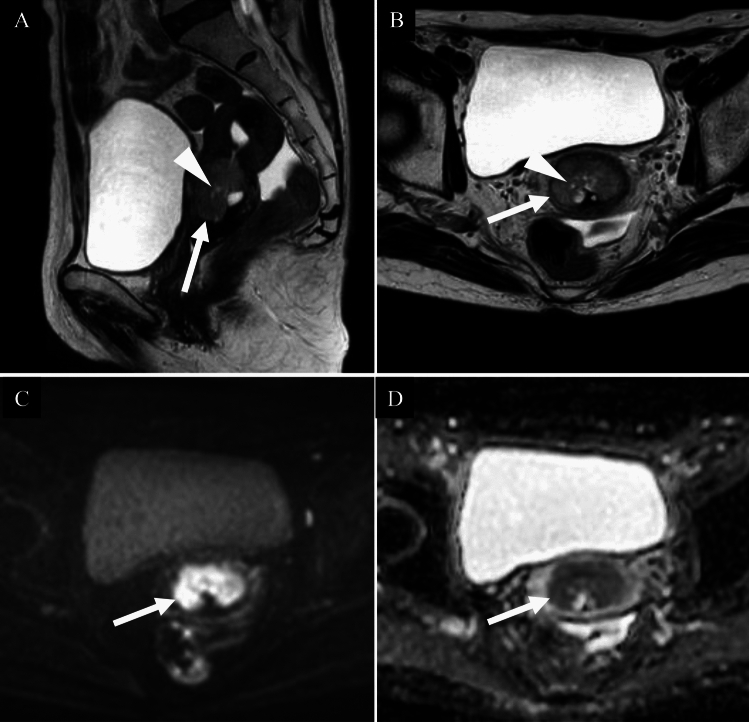
Gastric type adenocarcinoma of the uterine cervix in a woman in her sixties. **A** Sagittal T2-weighted image; **B** Axial T2-weighted image; **C** Axial diffusion-weighted image; **D** Axial apparent diffusion coefficient map. A T2-hyperintense mass is centered on the anterior wall of the uterine cervix and accompanied by small cysts (**A**, **B**: arrowheads) suggestive of gastric type adenocarcinoma (**A**, **B**: arrows). The lesion exhibits expansile growth with ill-defined margins. Although the mass shows a high signal intensity on diffusion-weighted image (**C**: arrow), the reduction in apparent diffusion coefficient values (**D**: arrow, ADC = 1.116 × 10⁻^3^ mm^2^/s) is less pronounced than that typically observed in squamous cell carcinoma, further supporting the diagnosis. The tumor was pathologically confirmed as FIGO 2018 stage IIB gastric type HPV-independent adenocarcinoma

MRI findings of GAS and LEGH often demonstrate either a “cosmos” (flower) [11, 12] or “microcystic” (raspberry) pattern [12, 13]. The cosmos pattern comprises small central cysts (< 5 mm) and solid components in the central part of the lesion, surrounded by larger peripheral cysts (≥ 5 mm)[11, 12], whereas the microcystic pattern features tightly clustered tiny cysts interspersed with solid and cystic areas [12, 13]. Distinguishing GAS from LEGH is challenging when a well-defined, massive, solid component is absent. LEGH often enlarges and more frequently exhibits a cosmos pattern with age, especially before menopause; both lesion size and pattern frequency tend to decrease after menopause [14]. Compared to usual type AC, GAS is more common in nulliparous and postmenopausal women and is less often diagnosed at FIGO stage I. GAS typically presents with ill-defined margins, endophytic or exophytic growth, intratumoural cysts or fluids, heterogeneous enhancement, and extensive involvement. GAS also shows greater tumor size and volume and higher apparent diffusion coefficient values than AC or SCC [9, 10, 15, 16] (Fig. 4). Notably, among patients with presumed early stage disease, the rate of preoperative underestimation is significantly higher in GAS [17].

### Adenocarcinoma, HPV-independent, clear cell type

**Fig. 5:**
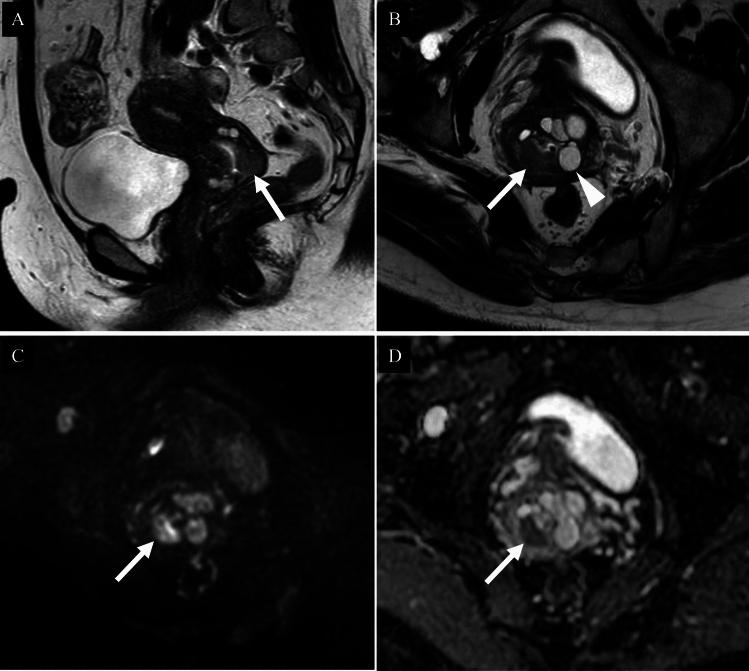
Clear cell carcinoma of the uterine cervix in a woman in her forties. **A** Sagittal T2-weighted image; **B** Axial T2-weighted image; **C** Axial diffusion-weighted image; **D** Axial apparent diffusion coefficient map. A T2-hyperintense mass is found to be localized to the posterior wall of the uterine cervix (**A**, **B**: arrows); the mass also shows restricted diffusion (**C**, **D**: arrows), with an ADC of 0.989 × 10⁻^3^ mm^2^/s. The surrounding cervical stroma is preserved. Multiple adjacent cysts are noted (**B**: arrowhead); however, these findings are consistent with those of Nabothian cysts rather than tumor-associated cysts. Differentiation from squamous cell carcinoma based on imaging findings alone is difficult. The tumor was pathologically diagnosed as an FIGO 2018 stage IB1 clear cell type HPV-independent adenocarcinoma

Clear cell carcinoma accounts for 2–4% of all ACs and is not associated with high-risk HPV infections. Histologically, it is a solid tumor with abundant glycogen-rich cytoplasm. Although clear cell carcinoma was previously associated with in utero exposure to diethylstilbestrol in young women (mean age, 19 years) [2], it is now rare and mostly sporadic, typically affecting older patients (mean age, 47 years) [2]. Clinically and prognostically, clear cell carcinoma resembles usual type AC, and imaging cannot reliably distinguish it from usual type AC or SCC [7] (Fig. 5).

### Adenocarcinoma, HPV-independent, mesonephric type

Mesonephric adenocarcinoma is a rare malignant tumor with mesonephric (Wolffian) differentiation that typically arises from remnants deep within the lateral cervical wall along the embryological mesonephric duct. It accounts for < 1% of all ACs and is not associated with high-risk HPV infection [2]. Mesonephric adenocarcinoma exhibits diverse histological patterns, and the preoperative diagnostic accuracy of biopsy is low (approximately 20%), often leading to misdiagnosis as more common ACs [18]. Although detailed imaging descriptions are lacking, distinguishing mesonephric AC from usual type AC or SCC based on imaging findings alone is difficult.

### Adenosquamous carcinoma

Adenosquamous carcinoma is a malignant epithelial tumor comprising squamous and glandular differentiation. It accounts for approximately 5–6% of all cervical cancers and is typically associated with high-risk HPV infection [2]. The average age at diagnosis is approximately 50 years [9]. Some studies have suggested that adenosquamous carcinoma may have a poorer prognosis than usual type AC [7, 19]. Compared with patients with SCC, patients with adenosquamous carcinoma tend to be younger and are more frequently diagnosed at FIGO stage I [9].

**Fig. 6:**
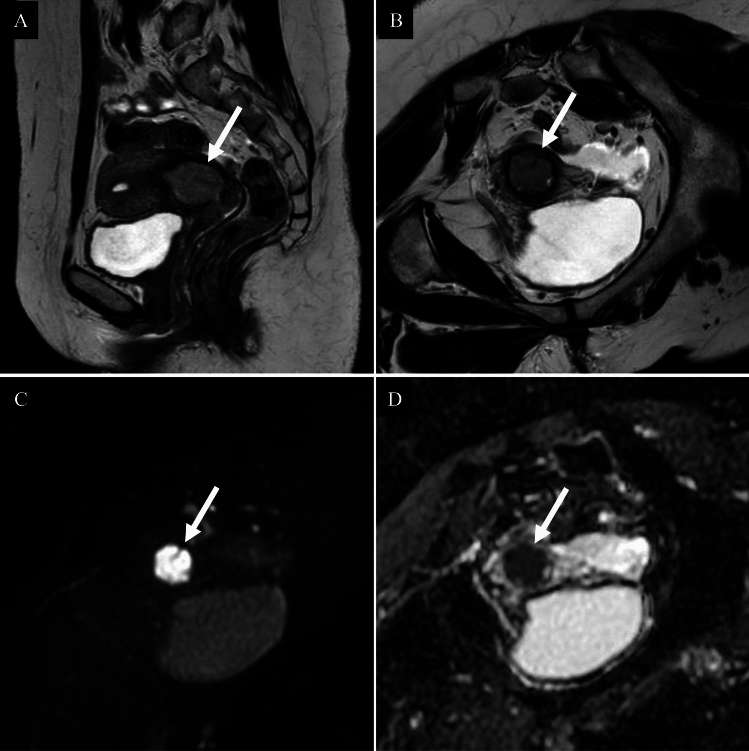
Adenosquamous carcinoma of the uterine cervix in a woman in her forties. **A** Sagittal T2-weighted image; **B** Coronal T2-weighted image; **C** Coronal diffusion-weighted image; **D** Coronal apparent diffusion coefficient map. A well-defined expansile cervical mass with high signal intensity relative to the surrounding stroma is observed on T2-weighted image (**A,** **B**: arrows). The lesion exhibits a markedly high signal on diffusion-weighted image (**C**: arrow) and significantly reduced apparent diffusion coefficient values (**D**: arrow), with an ADC of 0.728 × 10⁻^3^ mm^2^/s. No distinct imaging differences are noted when compared with squamous cell carcinoma. The surrounding cervical stroma appears to be preserved on T2-weighted image, with no evidence of parametrial infiltration. Pathological examination revealed vaginal wall invasion. The final diagnosis was FIGO 2018 stage IIA1 adenosquamous carcinoma

Mass formation is less common in adenosquamous carcinoma, and it often shows an infiltrative growth pattern, ill-defined margins, endophytic growth, intratumoural fluid or cystic components, and larger tumor volume [9] (Fig. 6).

### Common benign lesions as potential mimics

Cervical leiomyomas are rare compared to their uterine body counterparts, with an estimated frequency of 0.6% in hysterectomy specimens. This benign mesenchymal tumor, which is unrelated to high-risk HPV infection, shows smooth muscle differentiation with varying amounts of collagen-rich stroma [2]. On MRI, it typically presents as a well-defined solid mass, appearing hypointense relative to the myometrium on T2-weighted imaging, without diffusion restriction, in contrast to cervical cancer [7]. It may displace retroperitoneal structures, such as uterine vessels and ureters, reduce uterine mobility, and stretch the vaginal wall, complicating surgical management. Uterine artery embolization is a useful non-surgical alternative [20, 21].

Deep endometriosis, a common cause of pelvic pain and infertility, is defined as endometriotic lesions infiltrating subperitoneal structures and occasionally involving the cervix. On MRI, it typically appears as a solid tissue with low signal intensity on T2-weighted imaging and strong contrast enhancement, reflecting fibromuscular hyperplasia. High-signal foci on T1-weighted imaging may also be observed [22]. Polypoid endometriosis is a rare variant that mimics malignancy. It presents as a T2-hyperintense polypoid mass with signal characteristics similar to those of the endometrium and usually shows no diffusion restriction. Features such as the “black rim” sign and gradual contrast enhancement may support its benign nature [23]. However, endometriosis-associated malignancies can arise in this context and should be considered in the differential diagnosis [22, 24, 25].

Cervicitis refers to the inflammation of the uterine cervix. Although MRI is not routinely performed, non-specific retention cysts may be observed. These may appear as multicystic lesions with a high signal intensity on T1-weighted imaging because of secretions, proteinaceous content, or blood products [26]. Fibrosis can develop during healing, potentially leading to cervical stenosis. In acute cases, imaging features can resemble those of diffuse type GAS [27], and this possibility should be considered.

## Advances in imaging for diagnosis

### MRI

MRI is widely recommended as the first-line imaging modality for evaluating local tumor extent by all major international gynecologic oncology societies including the ESUR [28]. Recently, the potential applications of ultra-high b-values DWI at 3 T have been reported [29], and at 7 T, T2-weighted imaging-based evaluation has been explored [30].

**Fig. 7:**
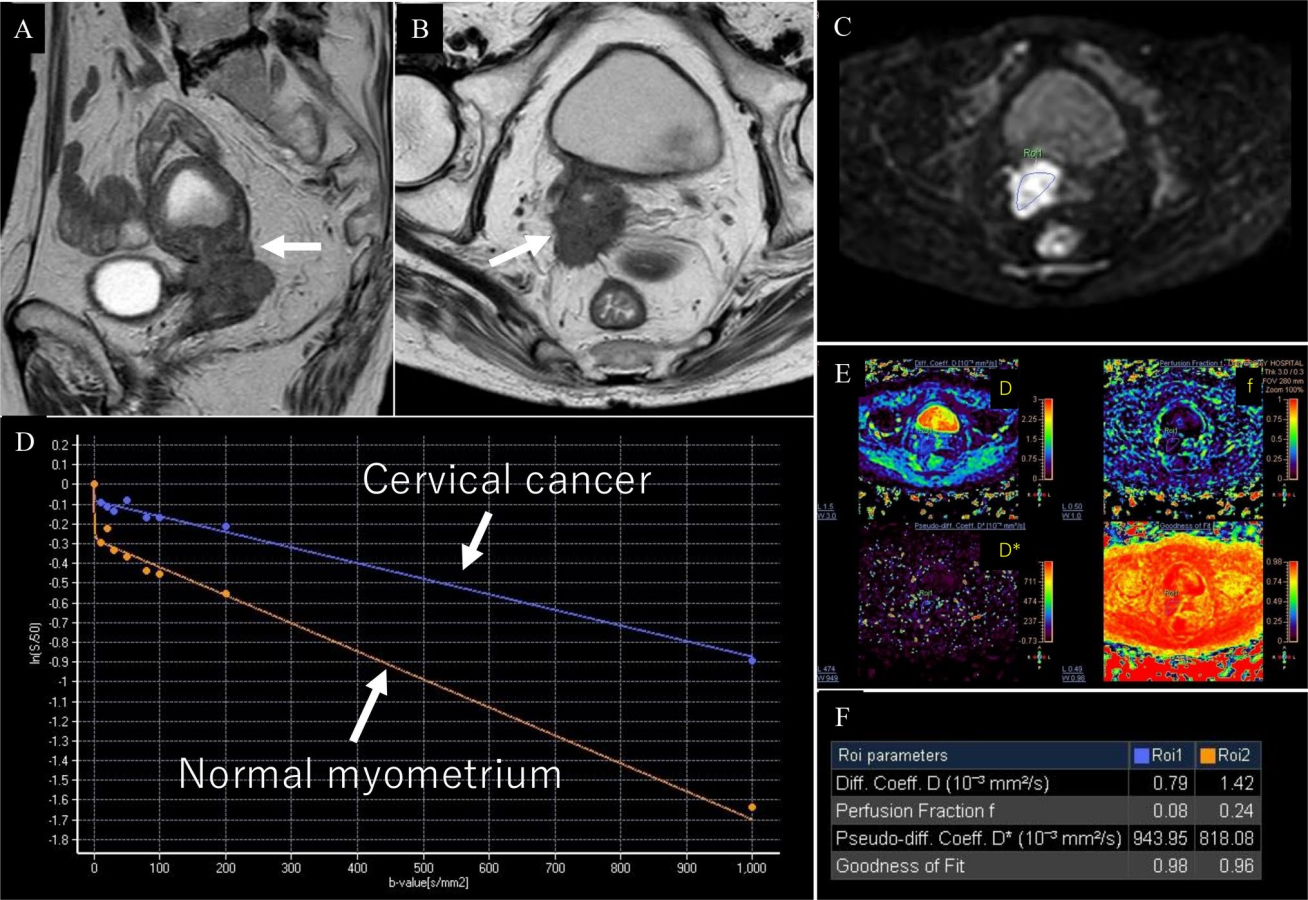
Intravoxel incoherent motion (IVIM) of a woman in her seventies with squamous cell carcinoma of the uterine cervix. **A** Sagittal T2-weighted image; **B** Axial T2-weighted image; **C** Axial diffusion-weighted image (b = 1000 s/mm^2^) and region of interest; **D** Bi-exponential fitting curves of IVIM signal decay, **E** Parametric maps of IVIM: diffusion coefficient (D), perfusion fraction (f), pseudo-diffusion coefficient (D*), and goodness-of-fit, **F**: Parameter values derived from IVIM. On T2-weighted imaging, a mass replacing the uterine cervix was observed, exhibiting serrated protrusions in all directions with definite parametrial invasion (**A**, **B**: arrows). IVIM reflects the signal attenuation plot of 9 b-values by separating the contributions of microperfusion and true molecular diffusion in the lower b-value range (blue curve: cervical cancer; orange curve: normal myometrium, **D**). In cervical cancer, compared with normal myometrium, D and f are reduced, whereas D* is increased, reflecting the high cellularity and abnormal tumor vasculature (**F**)

Multi-b-value DWI enables the calculation of advanced diffusion parameters based on intravoxel incoherent motion (IVIM) (Fig. 7) and diffusion kurtosis imaging (DKI), offering additional insights into tissue microstructure beyond those provided by conventional mono-exponential DWI analysis. IVIM reflects both tissue microperfusion and diffusion, whereas DKI captures the microstructural heterogeneity. In one study, the true diffusion coefficient, pseudo-diffusion coefficient, perfusion fraction, mean diffusivity, and apparent diffusion coefficient were all significantly lower, whereas the mean kurtosis was significantly higher in SCC than in AC [31]. In a previous study, the Ki-67 proliferation index was demonstrated to be a valuable biomarker for cervical cancer [32]. Texture analysis of IVIM using the true diffusion coefficient has been reported to be promising for predicting the Ki-67 proliferation index [33].

Pharmacokinetic analysis of DCE MRI quantifies the exchange of gadolinium-based contrast agents between the blood plasma and extracellular extravascular space [34]. This allows the calculation of parameters such as the volume transfer constant, extravascular extracellular volume fraction, blood plasma volume fraction, and efflux rate constant. A recent study reported that combining IVIM and DCE-derived parameters improved the diagnostic accuracy for detecting parametrial invasion in patients with cervical cancer [35].

Amide proton transfer-weighted imaging is based on chemical saturation transfer, which can noninvasively detect the exchange process between amide protons. This technique has been reported to be more effective than DWI and DKI in identifying histological grades of cervical cancer [36]. In contrast, another study concluded that both amide proton transfer-weighted imaging and DKI are useful; however, the former is advantageous for identifying the pathological type, grade, and stage [37].

Another study assessed the value of combining diffusion tensor imaging with IVIM for predicting parametrial invasion, showing that the true diffusion coefficient from IVIM and the fractional anisotropy from diffusion tensor imaging were useful indicators, and that the combined approach achieved superior diagnostic performance compared with either technique alone [38, 39].

In high-grade tumors, elevated T1 values on both native and contrast-enhanced T1 mapping have been reported, likely reflecting the increased collagen content and extracellular matrix changes associated with tumor progression. A study comparing native T1 values with DKI-derived parameters found that the difference between native and post-contrast T1, offered a discriminative value comparable to DKI in classifying tumor grade and identifying cervical cancer subtypes and stages [40].

### Positron emission tomography (PET)

2-deoxy-2-[^18^F]fluoro-D-glucose (FDG) PET/CT is recommended for patients with tumors ≥ 4 cm in size because of the increased risk of LN and distant metastases [41–45]. The enhanced sensitivity of PET/CT compared with that of CT alone is particularly advantageous for the detection of LN metastases [46].

Hybrid FDG PET/MRI is an emerging modality that combines anatomical details from MRI with molecular information from PET, enabling both quantitative and qualitative tumor assessment in a single session. It offers improved confidence in evaluating the cervical cancer extent and adjacent organ invasion, which may be challenging with PET/CT alone [47, 48]. PET/MRI also reduces radiation exposure, making it particularly suitable for younger patients and follow-up [47]. However, its superiority over the combination of separate pelvic MRI and FDG PET/CT beyond the convenience of a single session remains to be fully established [49].

In addition to FDG, various PET tracers have been investigated for their potential role in cervical cancer. Hypoxia PET, using tracers such as fluorine-labelled nitroimidazoles (e.g. [^18^F] FMISO) and copper-labelled diacetyl-bis (N-methylthiosemicarbazone) analogues (e.g. [^64^Cu] ATSM), enables non-invasive assessment of tumor hypoxia across space and time. [^64^Cu] ATSM offers several advantages, including rapid tumor uptake, faster clearance from normoxic tissues, and high target-to-background ratios, which may allow more accurate identification of hypoxic regions. Although promising, its effect on treatment planning and outcomes remains unproven [49]. Another promising approach is PET imaging with fibroblast activation protein inhibitors (FAPI), particularly [⁶⁸Ga]- or [^18^F]-labelled agents, which visualize tumor-associated desmoplasia, although its clinical utility in cervical cancer is still being explored [49].

### Artificial intelligence (AI) in imaging analysis

AI broadly refers to technologies that enable machines to perform tasks that mimic human intelligence. Machine learning, which is a subset of AI, allows systems to learn from data, whereas deep learning uses large neural networks to analyze complex patterns. AI-assisted models have shown promise in improving diagnostic accuracy and treatment monitoring [50]. Radiomics is an emerging field in which quantitative features are extracted from medical images using computational algorithms [51]. The integration of AI in radiomics, particularly machine learning and deep learning, has demonstrated considerable potential for advancing medical imaging. In cervical cancer, radiomics studies have most commonly focused on predicting LN metastasis [52–54] with others addressing lymphovascular space invasion [55] and prognosis [52, 56, 57]. While MRI is the predominant modality used, PET-based studies are also increasing [58, 59]. Direct image classification using deep learning is still limited [60], however, its application in image quality enhancement is growing [61–63]. AI -assisted automatic tumor segmentation　[64–67] plays a vital role in radiotherapy planning. Fragility fractures are a known complication of radiation therapy [68]. Recent studies have demonstrated the added value of AI in detecting pelvic and hip fractures, improving diagnostic accuracy [69, 70]. The development of large-scale publicly available imaging datasets is key to furthering AI research and enhancing patient care [71].

## Innovations in treatment strategies

### Surgical approaches

Recent robust evidence supports surgery as the standard treatment for early-stage cervical cancer, typically involving radical hysterectomy and pelvic lymphadenectomy. In stage IA1 (FIGO 2018) without lymphovascular space invasion, conization or simple hysterectomy can be performed without LN dissection. For IA2 (FIGO 2018), the European Society of Gynaecological Oncology (ESGO) [28] and the National Comprehensive Cancer Network (NCCN) guidelines [72] base treatment on lymphovascular space invasion status; simple conization or simple hysterectomy is recommended when it is absent, with optional sentinel LN biopsy. If lymphovascular space invasion is present, sentinel LN biopsy is recommended. When LN negativity is confirmed, radical hysterectomy, pelvic lymphadenectomy, and bilateral adnexectomy are performed with the surgical extent tailored to risk level [73]. Key prognostic factors in early stage disease include tumor size, lymphovascular space invasion, and depth of stromal invasion [46]. The simple hysterectomy and pelvic node assessment (SHAPE) trial found that simple hysterectomy offers similar pelvic recurrence rates at three years as radical hysterectomy in tumors ≤ 2 cm　[74]. In addition, the pelvic lymphadenectomy versus sentinel node biopsy using indocyanine green in early-stage cervical cancer (PHENIX) trial supports limiting pelvic lymphadenectomy when sentinel LN biopsy is successful, highlighting the shift toward less radical surgery [75]. In women of reproductive age with HPV-associated SCC or AC, ovarian preservation may be considered; however, it is not recommended for GAS [28]. Definitive concurrent radiotherapy is a valid alternative when risk factors predict adjuvant radiotherapy or when LN involvement is confirmed on imaging [28].

### Radiotherapy and brachytherapy

Concurrent chemoradiotherapy followed by brachytherapy remains the gold standard for the treatment of locally advanced cervical cancer. Weekly cisplatin combined with external beam radiotherapy improves both local control and five-year overall survival compared with radiotherapy alone. External beam radiotherapy typically delivers 45–50 Gy to the gross tumor and clinical target volumes with a boost to the involved LN. Advanced techniques, such as intensity-modulated radiotherapy and arc-based therapy guided by daily cone-beam CT, enhance precision, enable tumor monitoring, and reduce exposure to surrounding organs [47]. A randomized controlled trial conducted in 2024 demonstrated that adding pembrolizumab to concurrent chemoradiotherapy significantly improved progression-free and overall survival, particularly in FIGO 2014 stages IIIA, IIIB, and IVA [76]. This combination is now the preferred regimen according to the NCCN guidelines [77].

**Fig. 8:**
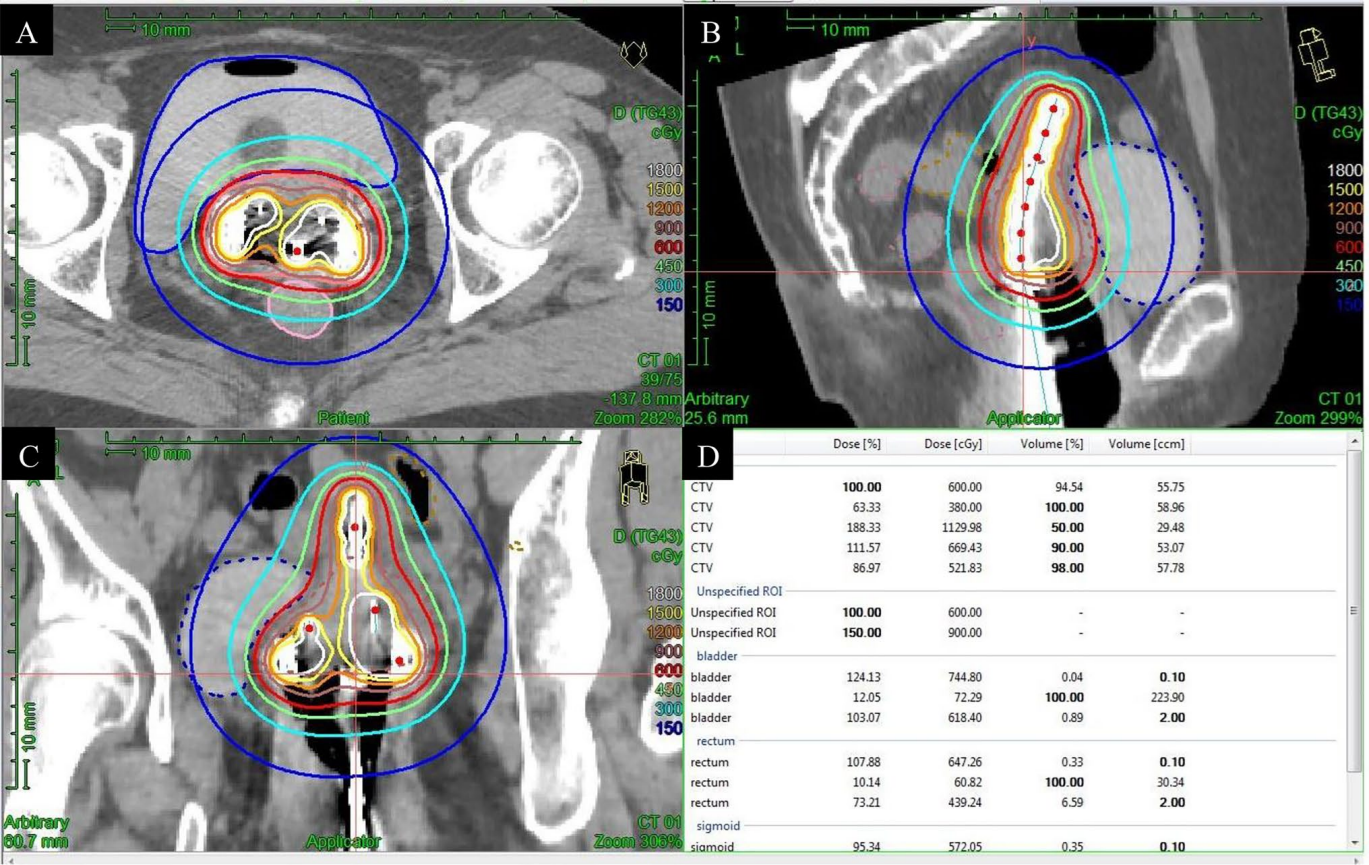
CT image-guided brachytherapy treatment plan visualization and dosimetry of squamous cell carcinoma in a woman in her sixties. The figure shows the dose distribution (cGy) surrounding the applicator in the **A** axial, **B** sagittal, and **C** coronal views. Table (**D**) summarizes the dose and volume parameters for the clinical target volume (CTV) and organs at risk. Key parameters included the dose [%] and dose [cGy] delivered to the specified percentage of the region of interest (ROI), as well as the volume [% and ccm] covered. Specific data is included for various CTV sub-regions (DTG43 and CASY) and organs at risk, including the bladder, rectum, and sigmoid colon

Brachytherapy remains essential after concurrent chemoradiotherapy and is usually performed using an intrauterine tandem and vaginal applicator. The approach depends on the tumor size and pelvic anatomy. If the tumor response rate exceeds 50%, intracavitary techniques may be sufficient; otherwise, interstitial brachytherapy is recommended. The shift to high-dose-rate brachytherapy has improved dose delivery and streamlined procedures. Image-guided brachytherapy using CT (Fig. 8) or MRI is now widely recommended to improve target coverage and reduce toxicity [47, 78]. Combined interstitial brachytherapy is being increasingly used, especially in Europe. In the image guided intensity modulated external beam radiochemotherapy and MRI-based brachytherapy in locally advanced cervical cancer (EMBRACE-I) trial, which validated image-guided adaptive brachytherapy, intracavitary or combined interstitial brachytherapy was applied in nearly half of the cases, achieving a 92% local control rate even in patients with modified FIGO 2009 stage IIIB disease, underscoring the benefit of technological advances in brachytherapy [79]. Combined interstitial brachytherapy via the transvaginal approach guided by transrectal ultrasonography has recently been reported to be both safe and effective [80]. Stereotactic body radiotherapy has shown promise for patients who are ineligible for brachytherapy [81]. In AC, which is generally more radioresistant than SCC, carbon-ion radiotherapy has demonstrated encouraging results owing to its high biological effectiveness [82].

**Fig. 9:**
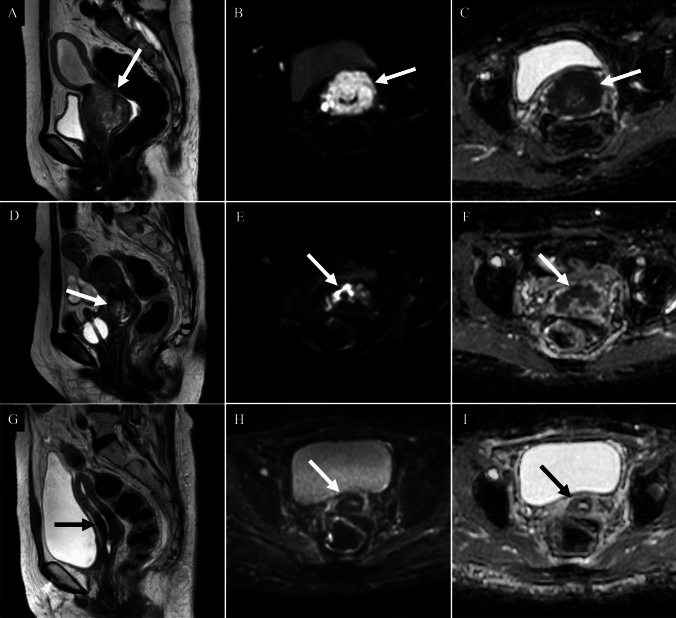
Squamous cell carcinoma of the uterine cervix in a woman in her 60 s treated with concurrent chemoradiotherapy. **A**, **D**, **G** Sagittal T2-weighted images; **B**, **E**, **H** Axial diffusion-weighted images; **C**, **F**, **I** Axial apparent diffusion coefficient (ADC) maps. **A–C** (before treatment): A bulky cervical mass with high signal intensity relative to the surrounding stroma is demonstrated on T2-weighted imaging (**A**: arrow), with marked diffusion restriction (**B**, **C**: arrows). The ADC was markedly reduced, with a mean value of 0.655 × 10⁻^3^ mm^2^/s. **D–F** (after 30 Gy pelvic irradiation): The tumor shows interval shrinkage on sagittal T2-weighted imaging (**D**: arrow). Diffusion-weighted imaging reveals residual high signal intensity in the right anterior cervical wall (**E**: arrow), corresponding to an area with an increased ADC of 1.018 × 10⁻^3^ mm^2^/s on the ADC map (**F**: arrow). **G**–**I** (a month after completion of all treatment, including intracavitary brachytherapy): Further regression is noted on sagittal T2-weighted imaging (**G**: arrow), with disappearance of the previously seen high signal on diffusion-weighted imaging (**H**: arrow). The ADC map (**I**: arrow) demonstrates a further increase in diffusivity, with a mean ADC of 1.436 × 10⁻^3^ mm^2^/s, consistent with complete response and reconstitution of the low-signal stromal ring

Pre-treatment MRI and FDG-PET/CT are recommended for radiation planning, while mid-treatment MRI enables brachytherapy dose adjustment according to residual tumor volume, improving local control and survival [41] (Fig. 9). Post-treatment MRI (including DWI) and FDG-PET/CT allow early detection and localization of residual disease, facilitating timely escalation to salvage therapy [41, 83–85]. DCE MRI provides prognostic information; tumors with poor perfusion typically show low enhancement before or early in concurrent chemoradiotherapy, which is associated with worse outcomes. Conversely, increased enhancement during treatment has been reported to indicate a better prognosis [41, 85]. Advanced imaging techniques, including quantitative parameters from DCE MRI, IVIM, PET/MRI, and radiomics, have also been reported to be useful for predicting local control [85–87].

### Fertility preservation treatment and treatment during pregnancy

Fertility-sparing treatment can only be performed in gynecologic oncology centers with comprehensive experience in this type of surgery. Each woman who wishes to preserve fertility and who has a histologic diagnosis of SCC or HPV-associated AC ≤ 2 cm should be informed about the possibility of fertility-sparing treatment [28]. The fertility-sparing treatment includes conization, simple trachelectomy, and radical trachelectomy. According to the ESGO 2023 guidelines, negative pelvic LN status is a prerequisite for any fertility-sparing treatment, for which sentinel LN biopsy is provided. The guidelines also emphasize that the primary objective should be resection of invasive tumors with sufficient free margins, while preserving the upper part of the cervix [28, 46]. The early-stage cervical cancer scheduled for fertility-sparing approach (ETERNITY) project is a retrospective, multi-institutional study that evaluated fertility-sparing treatment in early-stage cervical cancer. This study also investigated the safety of neoadjuvant chemotherapy and conization for tumors measuring > 2 cm in size. Among the 25 patients who underwent this approach, 16 (64%) completed the treatment, with three recurrences. Of the six patients who attempted conception, four (66.7%) achieved pregnancy [88, 89]. Accordingly, Pre-treatment MRI is mandatory to evaluate tumor diameter, the non-involved cervical length (upper tumor-free margin), the residual cervical length after conization, and the integrity of the low-signal stromal ring to exclude parametrial invasion [28]. The effect of radiotherapy on fertility preservation in adolescents and young adults has become an important consideration. Proton beam therapy and fertility-sparing techniques such as ovarian transposition and conservative surgery are increasingly emphasized in clinical guidelines [90]. Additionally, survivorship care must address long-term side effects including sexual dysfunction, lymphedema, and psychosocial concerns [91].

Cervical cancer is the most common gynecological malignancy diagnosed during pregnancy. Owing to its rarity, management should be centralized in specialized centers. The ESGO 2023 guidelines allow pregnancy preservation in early-stage disease (FIGO 2018 IA1–IB2) if LN involvement is excluded. In stage I cases, treatment may be delayed until fetal maturity, followed by a caesarean section with radical hysterectomy and lymphadenectomy [46]. Pregnancy termination is recommended if LN involvement is present. Platinum-based chemotherapy may be initiated at 14 weeks of gestation for locally advanced or residual tumors after conization. Vaginal delivery is associated with worse outcomes due to tumor dissemination and treatment delay, and therefore caesarean section after 32 weeks is preferred [46, 91].

## Systemic and immunotherapy approaches

Cisplatin-based chemotherapy, often combined with radiotherapy, remains the standard systemic treatment for advanced and recurrent cervical cancer. Recently, targeted therapies and immunotherapies have been investigated to improve patient outcomes. Monoclonal antibodies targeting epidermal growth factor receptor (EGFR), such as cetuximab and matuzumab, and tyrosine kinase inhibitors, such as gefitinib and erlotinib, have shown limited benefit in clinical trials [47, 92]. Among targeted agents, bevacizumab, an anti–vascular endothelial growth factor A (VEGF-A) antibody, has been shown to prolong survival when combined with platinum-based chemotherapy. However, its use is associated with increased risks of hypertension, thromboembolic events, and gastrointestinal fistulas [93]. Immune checkpoint antibodies, such as pembrolizumab (an anti–programmed cell death protein 1 [PD-1] antibody) and durvalumab (an anti–programmed death-ligand 1 [PD-L1] antibody), are currently under investigation in patients with recurrent or metastatic cervical cancer involving the PD-1/PD-L1 pathway. Early-phase trials have indicated that pembrolizumab is well tolerated and demonstrates modest clinical activity, with a progression-free survival of approximately 19 weeks and response rates ranging from 5 to 12% [94]. More recently, combination therapy with PD-1 antibodies and chemotherapy has emerged as a promising and effective treatment option for HPV-positive advanced cervical cancer [95]. Although these newer therapies are not yet the standard of care, they may offer alternatives for patients who are resistant to conventional treatment [92].

## Emerging technologies and future directions

Although HPV testing is well established for the diagnosis of cervical cancer, recent research has focused on identifying additional molecular biomarkers to predict treatment response and prognosis. Among protein biomarkers, elevated levels of VEGF and hypoxia-inducible factor-1 alpha (HIF-1α) have been associated with poor response to chemoradiotherapy, whereas lower levels are associated with better clinical outcomes. The SCC antigen is considered a marker of radioresistance and may indicate a higher risk of treatment failure. Recently, soluble intercellular adhesion molecule-1 (sICAM1) has emerged as a potential prognostic biomarker after radiotherapy [96].

Aberrant DNA methylation is another promising therapeutic target. Hypermethylation of genes such as PAX1, ZNF582, ESR1, and BRCA1 is commonly observed in cervical cancer and may suppress gene expression, making these genes predictive biomarker candidates. In addition, non-coding RNAs, particularly microRNAs, such as miR-9, miR-200a, and miR-145, have been implicated in the regulation of treatment response. Long non-coding RNAs and circular RNAs also have shown potential predicting treatment response and prognosis, although their mechanisms remain unclear [97].

Various therapeutic combinations are being explored, including a recent Phase Ib study evaluating the combination of durvalumab, cisplatin, and carbon-ion radiotherapy for locally advanced cervical cancer reported encouraging safety and efficacy outcomes, suggesting a novel therapeutic direction. Additionally, transarterial chemoembolization using cisplatin-loaded drug-eluting beads has been reported to be a potentially safe and effective treatment option for advanced or recurrent disease [98].

## Conclusion

Advances in molecular classification based on HPV status, imaging, artificial intelligence, and therapeutic strategies have transformed the paradigm of cervical cancer diagnosis and treatment. Open medical databases and AI-driven predictive models are expected to contribute to personalized care and improve clinical outcomes. The effective integration of these innovations into clinical practice, along with continued research efforts, will be the key to reducing mortality and enhancing patients’ quality of life.

## Data Availability

No datasets were generated or analyzed. Relevant information may be provided by the corresponding author upon reasonable request and subject to their discretion.
